# Effectiveness of Exercise-Based Cardiac Rehabilitation for Heart Transplant Recipients: A Systematic Review and Meta-Analysis

**DOI:** 10.1177/11786329231161482

**Published:** 2023-03-22

**Authors:** Rúben Costa, Emília Moreira, José Silva Cardoso, Luís Filipe Azevedo, João Alves Ribeiro, Roberto Pinto

**Affiliations:** 1 Faculty of Medicine, University of Porto, Porto, Portugal; 2 Department of Dermatology and Venereology, Centro Hospitalar Universitário de São João, Porto, Portugal; 3 CINTESIS, Centre for Health Technology and Services Research, Faculty of Medicine, University of Porto, Porto, Portugal; 4 RISE: Health Research Network; 5 Department of Medicine, Faculty of Medicine, University of Porto, Porto, Portugal; 6 Department of Cardiology, Centro Hospitalar Universitário de São João, Porto, Portugal; 7 Department of Community Medicine, Information and Health Decision Sciences, Faculty of Medicine, University of Porto, Portugal; 8 Faculty of Engineering, University of Porto, Porto, Portugal; 9 MIT Portugal Ph.D. candidate, Faculty of Engineering, University of Porto, Porto, Portugal; 10 Department of Biomedicine, Faculty of Medicine, University of Porto, Porto, Portugal

**Keywords:** Heart transplant, rehabilitation, high intensity interval training, systematic review, meta-analysis

## Abstract

**Background:**

Heart Transplant (HTx) is the ultimate chance of life for end stage Heart Failure (HF). Exercise training has consistently shown the potential to improve functional capacity in various chronic heart diseases. Still, the evidence in HTx recipients is scarcer. This study aims to systematically review the literature to evaluate the effectiveness and safety of Exercise-based Cardiac Rehabilitation (EBCR) in HTx recipients and to identify possible moderators of success.

**Methods:**

We conducted a systematic review and meta-analysis of randomized controlled trials on the effect and safety of EBCR in adult HTx recipients. The primary outcome was functional capacity, measured by Peak Oxygen Uptake (pVO2). We searched CENTRAL, MEDLINE, Embase, Scopus, and Web of Knowledge databases until December 2020, reviewed references of relevant articles and contacted experts. Usual care (UC), the different dosages of exercise regimens and alternative settings were allowed as comparators. A quantitative synthesis of evidence was performed using random-effects meta-analyses.

**Results:**

A total of 11 studies with 404 patients were included. Nine studies comprising 306 patients compared EBCR with usual care. They showed that EBCR improved pVO2 compared to usual care (Mean Difference [MD] 3.03 mL/kg/min, 95% CI [2.28-3.77]; *I*^2^ = 32%). In the subgroup analysis, including length of intervention and timing of enrollment after HTx, no significant moderator was found. Two trials, with 98 patients total, compared High Intensity Interval Training (HIIT) and Moderate Intensity Continuous Training (MICT). HIIT attained a significant edge over MICT (MD 2.23 mL/kg/min, 95% CI [1.79-2.67]; *I*^2^ = 0%). No major adverse events associated with EBCR were reported.

**Conclusion:**

We found moderate quality evidence suggesting EBCR has a significant benefit on functional capacity improvement HTx recipients at the short-term. HIIT showed superiority when compared to MICT. Research focusing long term outcomes and standardized protocols are needed to improve evidence on EBCR effectiveness.

## Introduction

Heart transplant (HTx) is the ultimate chance at life for many end-stage Heart Failure (HF) patients. Despite the significant survival benefit, heart transplant recipients frequently show limited functional capacity and poor quality of life (QoL) compared to the general population.^1-5^

One of the most relevant mechanisms explaining HTx patients’ functional limitation is the cardiac output deficit resulting from chronotropic incompetence in the denervated heart. Removing the parasympathetic stimulus results in a persistent higher heart rate at rest. Sympathetic denervation blunts the increased automaticity of the sino-atrial node induced by stress, limiting the heart’s capacity to achieve higher performances during exercise.^1,6^ Left ventricular diastolic disfunction also plays a role as highlighted by Squires and Bonikowske’s.^7^

Beyond cardiac allograft physiology mechanisms, peripheral factors, such as loss of strength and incompetent vasodilatory capacity also play a role. Most heart transplant recipients are profoundly deconditioned due to many years living with advanced heart failure and thus have poor exercise capacity and cardiac cachexia.^3,5,8-10^ Although many patients experience significant early improvement, the extent to which peripheral adaptations secondary to the chronic low output state are fully reversible is unknown. This in part helps to explain the reduced exercise arterial-mixed venous oxygen difference that HTx patients experience during exercise, which reflects tissues difficulties extracting oxygen from the blood.^7^

After transplant, patients’ health condition is also influenced by chronic immunosuppressive regimens, which affect muscular function and increase the risk of long-term complications such as cancer and renal failure. This, together with cardiac allograft vasculopathy (CAV) are responsible for many long-term complications and deaths.^11-13^

Exercise-based cardiac rehabilitation (EBCR) showed effectiveness in reverting peripheral alterations and improving oxidative capacity and capillary conductance, especially in the first year after heart transplant.^9,10^ At the long term, exercise rehabilitation may play an important role controlling cardiovascular risk factors.^10^ Both exercise and reinnervation improve hemodynamics, coronary blood flow and exercise thresholds, lowering resting heart rate and allowing peak heart rate to rise during exercise.^1,6^ It has also been hypothesized that exercise itself may accelerate this reinnervation, which could function as an auto-potentiation mechanism.^1,14,15^

Functional capacity constitutes a powerful prognostic factor in heart transplant patients as in the heart failure populations.^13,16^ Previous experiences have shown that rehabilitation in heart transplant is safe^1,15,17-19^ and beneficial. The results of the latest 2017 Cochrane meta-analysis indicated that the inclusion of these patients in cardiac rehabilitation programs was associated with exercise capacity improvement in the short term.^20^ Recently published retrospective studies have demonstrated that early rehabilitation after HTx is associated with a reduction in major adverse cardiovascular events in a dose-response fashion, a reduction in hospitalizations and long-term survival improvement.^9,18,21^

Historically speaking, heart transplant recipients have not been exposed to interval-based exercise with higher intensity, as it was considered dangerous due to delayed heart rate response. Nevertheless, high intensity interval training (HIIT) has gained notoriety due to growing evidence showing that this exercise modality may surpass the benefits of more traditional continuous moderate training regimens.^1,22-24^

Despite the accumulated knowledge and the recommendations from international societies,^25^ up-to-date guidelines with detailed exercise prescription for heart transplant recipients do not exist, and the use of EBCR is sub-optimal in this population. The present study aims to systematically review the literature to evaluate the clinical effectiveness exercise-based cardiac rehabilitation in heart transplant patients and to identify possible moderators of success.

## Methods

This systematic review and meta-analysis was implemented according to the Preferred Reporting Items for Systematic Reviews and Meta-Analyses (PRISMA) guidelines.^26^ The study protocol was registered in PROSPERO and can be consulted at CRD42021239110.

### Eligibility criteria

#### Study designs

This systematic review included randomized controlled trials (RCT) comparing exercise-based cardiac rehabilitation programs to usual care in the management of HTx recipients. It also comprised studies comparing different training modalities (eg, moderate intensity vs high intensity exercise training) and settings (eg, home-based vs center-based). Narrative reviews, preclinical studies, and editorial or opinion articles were excluded. Previous reviews and meta-analysis were assessed as guide, and reference lists were searched to identify additional RCTs.

#### Participants

We included studies examining interventions in adult HTx recipients with no restrictions regarding sex, ethnicity, and socioeconomic background at any time after the procedure.

#### Interventions

We defined EBCR as an intervention including physical exercise prescription by a cardiac rehabilitation specialist, performed at the hospital, cardiac rehabilitation center, at home or under a hybrid format including more than one location.^27^ High intensity interval training (HIIT) can be defined as interval durations of up to 4 minutes, with an intensity of ⩾85% of heart rate peak, ⩾80% Peak Oxygen Uptake (pVO2), or a Rating of Perceived Exertion (RPE) ⩾15, interspaced with up to 3 minutes active recovery intervals.^28,29^ Moderate-Intensity Continuous Training (MICT) includes continuous aerobic exercise of intensity 60% to 75% of heart rate peak, 50% to 65% of pVO2 or 12 to 15 RPE.^28,29^ To be considered for this study the intervention should last at least 4 weeks long and comprise an aerobic exercise modality.

#### Comparators

Usual care (UC) was defined as the standard management programs for adult heart transplant recipients, as proposed by the International Society for Heart and Lung Transplantation (ISHLT).^25^ This involves regular follow-up consultations to ensure the safety and optimal dosing of medicines and detect the development of complications or disease progression that may require a change in management.

#### Outcomes

The primary outcome of this review was (i) functional capacity and exercise tolerance, measured with pVO2 or 6-minute Walking Test. Secondary outcomes assessed were (ii) Adverse Events (All-cause mortality, cardiovascular mortality, All-cause Hospitalizations and cardiovascular Hospitalizations); (iii) Cardiac Allograft Vasculopathy; (iv) General and Disease Specific Quality of Life (QoL); and (v) Mental Health. As for inclusion criteria, studies should report a measure of functional capacity, either as primary or secondary outcome.

### Search strategy

We searched for studies meeting our eligibility criteria in 5 bibliographic databases: MEDLINE, SCOPUS, ISI - Web of Science, EMBASE, and Cochrane Central Register of Controlled Trials (CENTRAL) – our full queries are displayed in Supplemental Material 1. We have also performed manual searching through gray literature across clinicaltrials.gov to retain efficacy in the identification of additional published, unpublished, or ongoing trials. In addition, we browsed trial registers, contacted study authors, and searched the references of all relevant primary studies, as well as of other relevant systematic reviews. All studies published until December of 2020 were included. No limitation concerning language of publication was applied.

### Study selection

Two authors (RC and RP) independently screened the titles and abstracts of all records. Subsequently, relevant full-text articles were obtained and read by 2 independent authors. Inter-reviewer discrepancies were solved by discussion and consensus or by a third reviewer (EM) when agreement was not reached. When needed, authors were contacted regarding additional information for study eligibility assessment, or to request the full text when unavailable.

### Data extraction

Data was independently collected by 2 authors, using a standardized form. We retrieved data on study design, country, maximum follow-up length, inclusion and exclusion criteria, number randomized (for intervention and comparator), number for results (intervention and comparator), age, sex, intervention and comparator description and components, setting, frequency, intensity, exercise length, and time after transplant. Data regarding primary and secondary outcomes were also collected. Inter-reviewer disagreements were solved either by consensus or by a third reviewer when agreement could not be reached. Study authors were contacted to provide missing information.

### Quality assessment and publication bias

The systematic review and meta-analysis were performed using Review Manager (RevMan) 5.4. To assess the possible risk of bias (RoB) for each study, we collected information using the Revised Cochrane risk-of-bias tool for randomized trials (RoB 2).^30^ Each study was evaluated across 7 domains: random sequence generation, allocation concealment, blinding of participants and personnel, blinding of outcome assessment, incomplete outcome data, selective reporting, and other bias. Studies were categorized as overall “high risk,” “low risk,” or “unclear risk” of bias with the help of RoB 2. Studies with 3 or more individual domains categorized as unclear risk or 1 high risk domain were classified as “overall high risk.” We computed graphic representations of potential bias within and across studies using RevMan. Assessments of overall quality of evidence were performed using the GRADE approach for the primary outcome.

### Data synthesis

Random-effects meta-analysis regarding functional capacity (measured with pVO2) was performed comparing EBCR with UC (primary analysis) and HIIT versus MICT (secondary analysis). The Mean Difference (MD) was used as the effect measure. For the studies reporting values of mean and standard deviation (SD) at the beginning and end of intervention both for experimental and control groups, a new mean and SD was calculated using the difference between the final and initial measurements and adjusting for the paired nature of this comparison. A similar adjustment was considered for a crossover trial used in a separate comparison of HIIT versus MICT. Those values were then used to calculate the difference between the experimental and control group and respective standard error, which is presented in the secondary analysis. Correlation between final and initial measurements was calculated from the studies providing SD at baseline, follow-up, and change score. When no information regarding SD was reported, we contacted authors for full description of results. In case of no further information, a mean of the remaining standard deviations was imputed in the analysis. Heterogeneity was analyzed using the Cochran’s Q statistic, the Chi-square test and I-square statistic (*I*^2^). Moderate or severe heterogeneity was considered if I^2^ > 50% and I^2^ > 75%, respectively. To assess potential moderators of heterogeneity in the primary analysis, the following subgroup analysis were performed: time after heart transplant, intervention length, resistance training (RT) inclusion, overall risk of bias, setting, and aerobic training intensity.

A qualitative description was performed for other outcomes that could not be included in the meta-analysis.

## Results

### Study selection

**Figure 1.**
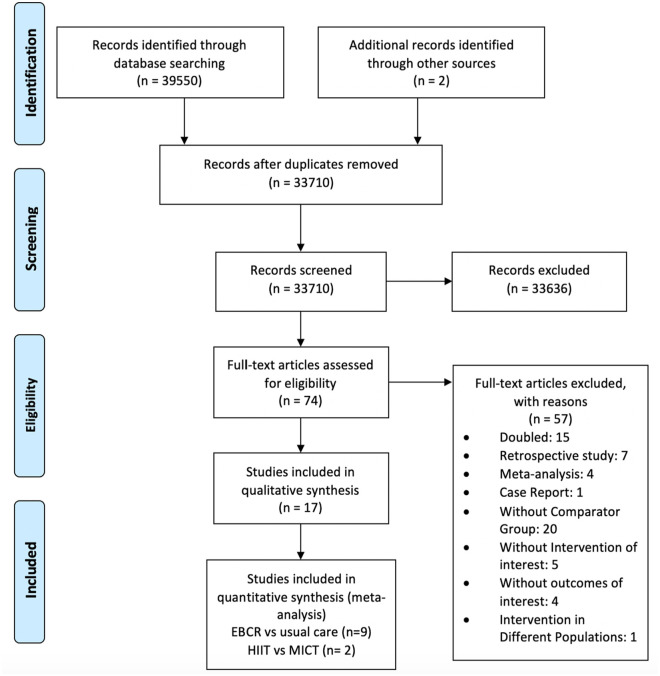
Flowchart of included studies.

Out of the 39 552 records initially identified, 11 studies were included in the meta-analyses: 9 studies comparing exercise-based cardiac rehabilitation with usual care,^14,15,31-37^ 2 studies comparing High Intensity Interval Training and Moderate Intensity Continuous Training.^23,38^ The selection process and reasons for exclusion are further detailed in Figure 1.

### Studies and patients’ characteristics

A total of 11 studies with 404 patients (mean age 52 years) were included in 2 separate meta-analysis. For the primary meta-analysis 306 patients from 9 studies comparing EBCR versus UC,^14,15,31-37^ and 98 patients from 2 studies comparing HIIT with MICT.^23,38^ Only one study with 40 patients compared home-based and center-based settings.^39^ This study was naturally not depicted with meta-analytical variables as it was an isolated investigation. In all trials, most patients were male. In all studies functional capacity was reported as pVO2.

One study^40^ was excluded from the analysis due to concerns regarding patients matching with Tegtbur et al^36^ study. An email was sent to address this issue, but no answer was provided.

Further detailed information regarding each study included in both meta-analysis is outlined in Table 1. More specific information regarding studies only included in qualitative synthesis is available as Supplemental Material 2.

**Table 1.** Characteristics of individual studies (RCTs) included in both meta-analysis.

First author, country	Enrolled participants (Int vs Ctrl)	Time of recruitment after transplant	Setting	Intervention	Control	Frequency (days per week)	Intervention length (M)/follow-up length (M)
Kobashigawa et al,^37^ USA	14 vs 13	Within 2 week	CB	MICT + RT	UC	1-3 initially, then reduced to 1 day every 2 weeks.	6/6
Tegtbur et al,^36^ Germany	16 vs 15	Mean 5.1 ± 2.2 years	HB	MICT	UC	Once every 2 days.	12/12
Bernardi et al,^14^ Italy	13 vs 11	6 months	HB	MICT	UC	5	6/6
Wu et al^34^, Taywan	18 vs 19	Minimum 12 months	HB	MICT + RT	UC	3	2/2
Braith et al,^35^ USA	10 vs 10	2 months	CB	MICT + RT	UC	3	3/3
Haykowsky,^33^ Canada	22 vs 21	At least 6 months	CB	MICT + RT	UC	5	3/3
Hermann et al,^32^ Denmark	15 vs 15	At least 12 months	CB	HIIT	UC	3	2/2
Nytrøen et al,^15^ Norway	26 vs 26	1-8 years	CB	HIIT	UC	3	6/12
Dall et al,^38^ Denmark	17	Minimum of 12 months	CB	HIIT	MICT	3	3/11
Pascoalino et al,^31^ Brazil	33 vs 9	At least 12 months	Hybrid	MICT	UC	3	3/3
Nytrøen et al,^23^ Scandinavia	39 vs 42	Mean of 11 weeks	CB	HIIT	MICT	3	9/12

Abbreviations: CB, center-based; Ctrl, control group; HB, home-based; HIIT, high intensity interval training; Int, intervention group; M, months; MICT, moderate intensity continuous training; RT, resistance training; UC, usual care.

### EBCR characteristics

In most studies (n = 8), patients were recruited 6 or more months after transplant.^14,15,31-34,36^ Overall, 7 trials performed center-based exercise (CB),^15,23,32,33,35,37,38^ 3 studies performed home-based exercise (HB),^14,34,36^ 1 was a hybrid regimen (HB and CB).^31^ In most of the studies frequency of training was 3 sessions per week. The length of session ranged from 28 to 52 minutes with a median length of 30-minutes. None of the studies had an intervention that lasted longer than a year. One study compared HB and CB^39^ and it consisted in 8 weeks of MICT and RT performed 3 times per week.

### Risk of bias assessment

Overall bias classification was high risk. Out of the 11 studies, 4 were classified as overall unclear risk^23,31,32,38^ and 7 as overall high risk.^14,15,33-37^

Selection bias related to random sequence generation was low risk in almost all trials. Three studies^14,35,36^ were classified as unclear risk due to vague description of the randomization process. Two were deemed as high risk due to imbalance of the groups at baseline.^33,34^ Allocation concealment was only described in 3 studies.^23,32,38^ The remaining 8 studies were classified in the unclear risk category because we were unable to find if concealment of intervention and control groups was initially done.

All studies were classified as unclear risk of bias for performance bias because of the inherent problem of blinding participants and personnel staff in exercise interventions. Considering detection bias the most frequent result was unclear risk, with 8 studies not clarifying this point.^14,15,23,33-37^ Only 3 were classified as low risk of bias since blind assessment was performed.^31,32,38^

Regarding attrition bias 8 studies were categorized as low risk^14,15,23,31-33,37,38^ and 3 studies^34-36^ were classified as high risk because there were patient losses to follow-up sufficient enough to dictate this classification. As for selective reporting all studies were categorized as low risk.

**Figure 2.**
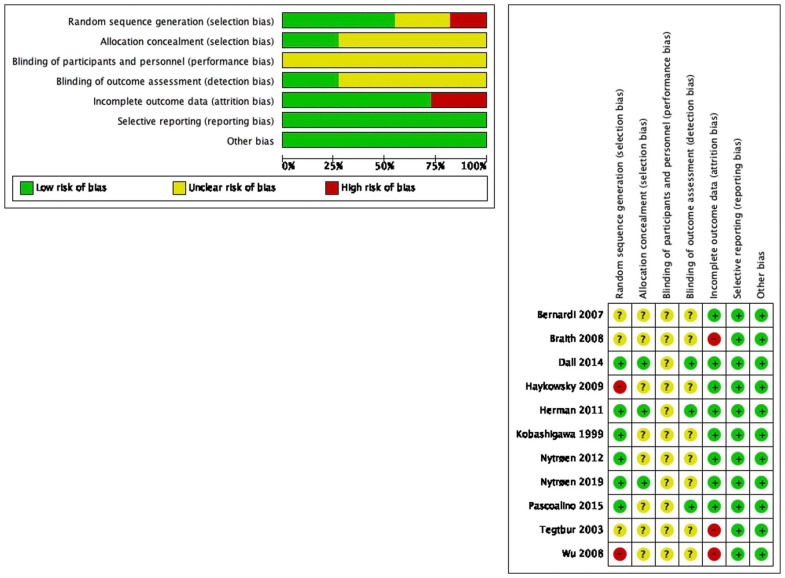
Risk of bias graphics.

The graphs in Figure 2 schematically represent the bias assessment.

### Functional capacity

#### EBCR versus UC

**Figure 3.**
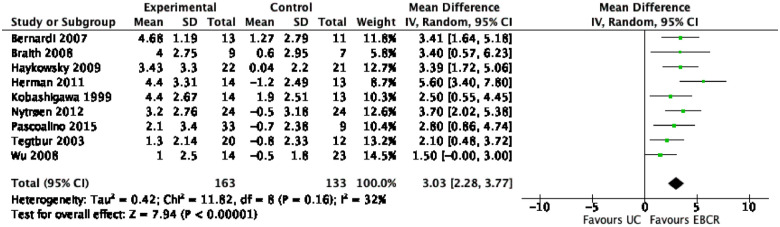
Forest plot of the primary analysis: Exercise based cardiovascular rehabilitation (EBCR) versus usual care (UC).

Nine studies comprising 306 patients compared exercise EBCR with UC.^14,15,31-37^ Patients randomized to EBCR showed higher improvement in pVO2 than those in UC (MD 3.03 mL/kg/min, 95% CI [2.28-3.77]). Low heterogeneity was found (*I*^2^ = 32%) (Figure 3). GRADE assessment considered evidence as moderate.

In the sub-analysis regarding the potential moderators of intervention success – time after heart transplant (<6 or ⩾6 months), intervention length (⩽3 or >3 months), resistance training inclusion, overall risk of bias assessment, setting (home-based, center-based or hybrid) and intensity of aerobic exercise (HIIT or MICT) – none of these variables explained significantly (*P* > .05) the gain in pVO2 of EBCR over UC. Nevertheless, we observed an increase in the mean difference of pVO2 of at least 1 mL/kg/min when considering HIIT studies and unclear risk of bias compared to MICT and high risk of bias, respectively (Supplemental Figures 1–6).

#### HIIT versus MICT

**Figure 4.**
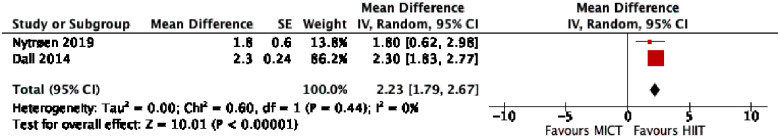
Forest plot of the secondary analysis: High intensity interval training (HIIT) versus moderate intensity continuous training (MICT).

Two studies comprising 98 patients compared the impact of HIIT versus MICT on pVO2 change.^23,38^ Dall et al^38^ consisted in a crossover trial with 17 patients with a 5-month washout period in between, each arm performing 12 weeks of exercise. Nytrøen et al performed a 9-month intervention study with 81 patients recently transplanted. Patients in HIIT group showed a higher improvement in functional capacity compared to MICT group (MD 2.23 mL/kg/min, 95% CI [1.79-2.67]). Low heterogeneity was found (*I*^2^ = 0%) (Figure 4). GRADE assessment considered evidence as moderate.

#### CB versus HB

Karapolat et al^39^ compared the differential effect regarding CB versus HB intervention in a group of 40 patients. Patients in the CB group showed a substantial improvement of pVO2 between baseline and follow-up (16.73 ± 3.91 mL/kg/min vs 19.53 ± 3.89 mL/kg/min, *P* = .002), compared with no difference in HB group patients (20.12 ± 4.40 mL/kg/min vs 19.48 ± 4.53 mL/kg/min, *P* = .36). The difference between groups was significant (*P* = .01).

#### Maintenance of effect

One study comparing EBCR versus UC^41^ and 2 studies comparing HIIT versus MICT^38,42^ analyzed the maintenance of effect on pVO2 after intervention cessation.^38,41,42^

Yardley et al^41^ conducted a 5-year follow-up of 21 patients in EBCR and 20 patients in UC, included in Nytrøen et al^15^ study. Mean change at 5-year follow-up was −1.75 [−3.83; −0.33] in EBCR and −2.78 [−5.32; −0.25] in UC. The authors concluded that at 5-year follow-up the EBCR had lost its beneficial impact when compared to UC.

Dall et al^38^ reported the 5-month period wash-out in the crossover study. Patients were told to resume normal activity. Functional capacity, assessed by pVO2, dropped significantly both in HIIT (n = 8) (27.4-23.6 mL/kg/min) and MICT (n = 8) (23.0-20.9 mL/kg/min). Aggregating both groups (n = 16) pVO2 dropped significantly from 26.3 ± 7.0 to 23.4 ± 6.7 mL/kg/min (*P* < .001).

Rolid et al^42^ re-analyzed the patients studied in Nytrøen et al^23^ in a 3-year follow-up study. In the HIIT group 79% of patients exercised ⩾2 weeks versus 82% in the MICT group. Functional capacity remained stable with a small decline in pVO2 in both groups: −0.3 mL/kg/min in the HIIT group versus −0.9 mL/kg/min in the MICT group (p for difference = 0.497). Thus, the beneficial impact of HIIT over MICT at 1 year follow-up reported by Nytrøen 2019 was lost.^23,42^

#### Adverse events

None of the included studies reported on all-cause mortality or cardiovascular mortality. Nytrøen et al^15^ reported a myocardial infarction in the usual care group which forced this patient to withdraw but none in the intervention group. The remaining studies did not report considerable adverse events. The rate of the events was similar in HIIT and MICT groups and the incidence and nature of the events were similar to the general HTx population.

#### Cardiac allograft vasculopathy

Only one study^43^ reported the effects of EBCR in cardiac allograft vasculopathy (CAV). Nytrøen et al^15^ performed a sub-study of their HIIT trial cohort using serial intravascular ultrasound measurements. Progression of cardiac allograft vasculopathy was reduced by more than 50% in the HIIT group compared to the control group, as measured by a smaller mean increase (Δ [95% CI]) in percent atheroma volume (PAV): 0.9% [−0.3% to 1.9%] versus 2.5% [1.6% to 3.5%] (*P* = .021), Δ total atheroma volume (TAV): 0.3 [0.0-0.6] mm^3^/mm versus 1.1 [0.6-1.7] mm^3^/mm (*P* = .020), and Δ total plaque volume: 8.0 [0.3-15.7] mm^3^ versus 25.6 [14.8-36.4] mm^3^ (*P* = .011).

#### Quality of life

The diversity in the evaluation methodologies used to measure QoL, as well as missing data, limited the implementation of meta-analysis.

Three studies comparing EBCR versus UC,^15,34,36^ 3 studies comparing HIIT with MICT at 1-year follow-up and 3-year follow-up^23,38,42,44^ reported data regarding the impact of the interventions on QoL.

Studies comparing EBCR versus UC evaluated QoL using Short Form 36 version 2 (SF-36), Profile of Quality of Life in the chronically ill (PLC), World Health Organization Questionnaire on Quality of Life (WHOQoL-BREF), and Visual Analog Scale (VAS).

Only Wu et al^34^ provided baseline and follow-up results in the WHOQoL-Bref, and respective mean differences. Even though patients in EBCR group showed higher improvements in QoL domains compared to UC group, these differences were not statistically different.

Nytrøen et al^15^ used the Short Form 36 version 2 (SF-36), standardized Physical Component Summary (PCS), and Mental Component Summary (MCS) from the previous and VAS to compare those who exercised to those who did not. Patients in EBCR group showed higher improvement compared to UC in the General health domain (mean 54 vs 49, *P* < .05) and VAS (mean 65 vs 26, *P* < .05). All the remaining domains were non-significant.

Tegtbur et al^36^ used the Profile of Quality of Life in the chronically ill (PLC). Results were graphically reported and showed a beneficial impact in the physical function domain (*P* < .05) and in the physical well-being domain (*P* < .01) favoring exercise. All the remaining domains were non-significant.

In studies comparing HIIT versus MICT, both interventions did not differ significantly in the improvement in QoL domains. SF-36 and VAS were the scales used.

Dall et al^20,38^ used SF-36 and the comparisons between HIIT and MICT across all domains were non-significant. Nytrøen et al^23,44^ made use of the PCS and MCS scores of the SF-36 and VAS. Neither HIIT nor MICT could show advantage to one another at 1-year follow-up. Rolid et al^44^ analyzed the same cohort of patients but with a 3-year follow-up. The results were also non-significant for PCS, MCS, and VAS.

A table which compares the studies reporting QoL is available as Supplemental Material 3.

#### Mental health

Overall, 3 studies reported data on mental health. Christensen et al^45^ reported information using the Hospital Anxiety and Depression Scale (HADS), regarding the patients present at Hermann et al’s^32^ study. Anxiety score (HADS-A) decreased significantly in the exercise group (4.7 ± 1.8 to 1.8 ± 0.8, P = .01) but not in the control group (3.2 ± 1.6 to 3.7 ± 2.3, P = NS). No impact was reported on depression score (HADS-D).

Yardley et al^41^ reported the difference at baseline and at 5-year follow-up for some of those patients enrolled in Nytrøen et al.^15^ Anxiety assessed by the HADS-A questionnaire decreased in the HIIT group and increased in the control group with a significant difference at the 5-year follow-up (*P* = .01). There were no differences between groups when comparing the depression scores at 5-year follow-up.

In Nytroen et al^23^ no impact was reported regarding HIIT versus MICT on HADS-A or HADS-D scores.

## Discussion

In this systematic review we have analyzed the effectiveness of EBCR in the follow-up of HTx patients. Overall, 11 primary studies were identified, including 404 patients, 9 for the EBCR versus UCR and 2 studies for the HIIT and MICT comparison. We found moderate quality evidence that EBCR in HTx recipients leads to improvement in functional capacity in short-term follow-up (less than a year) compared to UC alone.

We also found moderate quality of evidence that HIIT is feasible, safe, and superior to MICT at improving functional capacity in this population at the short-term. Importantly, this benefit may be lost at the long term^42^ reinforcing the uncertainty of HIIT long-term benefits compared with MICT.

Regarding the impact of EBCR at the long term, Yardley et al^41^ and Dall et al^38^ allowed us to capture one of the Achilles tendon of cardiovascular rehabilitation. Interventions must be kept regularly to provide a benefit. 4 years after cessation of the HIIT intervention 41 of 52 patients enrolled in Nytrøen et al’s^15^ trial performed cardiopulmonary evaluation only to realize that the initial 3.6 mL/kg/min superiority of EBCR was lost. Dall et al^38^ study is even more dramatic. In a period of just 5 months, the pVO2 of 16 exercising patients (half performed HIIT and the other half MICT) dropped from 26.3 to 23.4 mL/kg/min. This shouts attention to the need of these patients to continue to exercise regularly and the urgency of effective strategies for this to be accomplished.

Only one study^39^ compared head-to-head the setting of the intervention. The center-based approach reached meaningful improvement when compared to the home-based intervention. However, this result should be interpreted with caution, since patients in the home-based exercise group utterly failed to raise their functional capacity. Studies by Tegtbur et al^36^ and Bernardi et al^14^ conducted home-based experiences and shown that patients were able to significantly improve their exercise capacity. In subgroup analysis including only studies with HB, pVO2 still improved meaningfully. As many of HTx recipients live at long distances from the transplant center, HB programs are of particular relevance in this population and the message should be on to put the majority of HTx patients exercising, at home or at a rehabilitation center.

Few studies reported on adverse events. None reported prognostic benefit. This may be related to the short follow-ups. Peak Oxygen Uptake is a well-documented powerful prognosticator in those with chronic cardiovascular conditions but the evidence addressing the relation of pVO2 and survival in heart transplant recipients is scarcer.^46^ Retrospective data^16^ suggests that pVO2 is associated with long-term survival in HTx recipients. A more recent retrospective study^47^ has shown that participation in cardiac rehabilitation was associated with a 29% reduction in 1-year readmissions. So, the finding of 3.03 mL/kg/min improvement in pVO2 is relevant not only for the improved capability of patients, but also for what it can mean prognostic wise. However, this should be confirmed in long-term trials.

Only one study investigated the effects on cardiac allograft vasculopathy (CAV)^43^ showing a reduction of 50% in progression as measured by intravascular ultrasound. The pathophysiological process of CAV is complex and involves many factors, including innate and adaptive immune responses as well as traditional risk factors. The way by which exercise affects CAV progression is not fully understood. Theoretically, this may result in part from the reduction in pro-inflammatory state. Nevertheless, it is not yet fully understood to what extent the effect on CAV depends on the HIIT effect, such that this finding needs to be confirmed in future studies.

We did not find any consistent effect of EBCR on QoL. This can relate to different reporting methods, which precludes aggregation of data, the relativity low number of patients, and the inherent problematic quality of the trials. HIIT demonstrated a beneficial impact on anxiety at both short^32,45^ and long-term.^41^ Caution should be taken since we are discussing isolated reports.

### Strengths and limitations

This systematic review is important as it is the first to shed some light on the longer follow-up studies in the heart transplant recipient’s field. Its importance also comes from the fact that it compares HIIT and MICT interventions besides the traditional comparison of EBCR and UC. This is also, that we are aware, the first meta-analysis using the mean differences of pVO2 values attained during the rehabilitation. This matters because takes into consideration the baseline fitness levels of the individuals and enables us to see a more precise estimate of the real impact of exercise in HTx populations. Other strong asset of this review is the query designed who allowed us to reach 39 550 references and the rigorous methodology implemented. We believe this study to be the most comprehensive and up to date meta-analysis in HTx rehabilitation.

Major impediments for stronger evidence are the relatively low number of patients, the scarcity of trials (only 9 studies for the primary analysis and 2 studies for the HIIT vs MICT comparison) and the short follow-up length. In fact, this meta-analysis included only one more trial^23^ than the latest Cochrane meta-analysis published,^20^ which reflects the lack of investigation on the field. Other limitation is the suboptimal quality of the studies retrieved and the lack of standardization in the exercise regimens across studies. Most studies included in the meta-analysis began exercising patients many months which could represent an external validity problem, as the majority of real-life programs commence in the first month post-transplant. So, as of today, and because of these limitations, we can only say that exercise improves short-term functional capacity (less than a year). Longer follow-up studies are needed to see the real impact of a one-time intervention and, perhaps, to arrange and ideal timing for a repeated structured program. Another particularly important limitation that proceeds from the short-term follow-up is the nonexistence of prospective randomized data on mortality and hospitalizations.

## Conclusion

We found moderate quality evidence suggesting EBCR has a significant benefit on functional capacity improvement in HTx recipients at the short-term. HIIT showed a slight superiority when compared to MICT. To sustain a prolonged effect, HTx recipients need to continue exercising. This may be facilitated by HB or hybrid programs. Further research with focus on long-term benefits and more standardized protocols are needed to build more robust evidence on EBCR effectiveness in this population.

## Supplemental Material

sj-docx-1-his-10.1177_11786329231161482 – Supplemental material for Effectiveness of Exercise-Based Cardiac Rehabilitation for Heart Transplant Recipients: A Systematic Review and Meta-Analysis Click here for additional data file. Supplemental material, sj-docx-1-his-10.1177_11786329231161482 for Effectiveness of Exercise-Based Cardiac Rehabilitation for Heart Transplant Recipients: A Systematic Review and Meta-Analysis by Rúben Costa, Emília Moreira, José Silva Cardoso, Luís Filipe Azevedo, João Alves Ribeiro and Roberto Pinto in Health Services Insights

sj-docx-2-his-10.1177_11786329231161482 – Supplemental material for Effectiveness of Exercise-Based Cardiac Rehabilitation for Heart Transplant Recipients: A Systematic Review and Meta-Analysis Click here for additional data file. Supplemental material, sj-docx-2-his-10.1177_11786329231161482 for Effectiveness of Exercise-Based Cardiac Rehabilitation for Heart Transplant Recipients: A Systematic Review and Meta-Analysis by Rúben Costa, Emília Moreira, José Silva Cardoso, Luís Filipe Azevedo, João Alves Ribeiro and Roberto Pinto in Health Services Insights

sj-docx-3-his-10.1177_11786329231161482 – Supplemental material for Effectiveness of Exercise-Based Cardiac Rehabilitation for Heart Transplant Recipients: A Systematic Review and Meta-Analysis Click here for additional data file. Supplemental material, sj-docx-3-his-10.1177_11786329231161482 for Effectiveness of Exercise-Based Cardiac Rehabilitation for Heart Transplant Recipients: A Systematic Review and Meta-Analysis by Rúben Costa, Emília Moreira, José Silva Cardoso, Luís Filipe Azevedo, João Alves Ribeiro and Roberto Pinto in Health Services Insights
