# Supplementary material for: Effectiveness of Exercise-Based Cardiac Rehabilitation for Heart Transplant Recipients: A Systematic Review and Meta-Analysis
Source: Health Serv Insights. 2023 Mar 22;16:11786329231161482. doi: 10.1177/11786329231161482 (PMC10034295; doi:10.1177/11786329231161482)
Supplement: sj-docx-1-his-10.1177_11786329231161482 – Supplemental material for Effectiveness of Exercise-Based Cardiac Rehabilitation for Heart Transplant Recipients: A Systematic Review and Meta-Analysis [file sj-docx-1-his-10.1177_11786329231161482.docx]

**Online Supplementary Material 1:** Search executed in December 2020 in all the databases

**MEDLINE**

1. MeSH descriptor: [Heart transplantation] explode all trees

2. ("heart transplant" OR "heart transplant recipient" OR “heart graft” OR “cardiac transplant” OR “heart recipient” OR “heart transplant”)

3. (#1 OR #2)

4. MeSH descriptor: [Cardiac Rehabilitation] explode all trees

5. MeSH descriptor: [Rehabilitation] explode all trees

6. MeSH descriptor: [Exercise] explode all trees

7. MeSH descriptor: [Exercise therapy] explode all trees

8. MeSH descriptor: [Sports] explode all trees

9. MeSH descriptor: [Physical Exertion] explode all trees

10. MeSH descriptor: [Physical Education and Training] explode all trees

11. ("Exercise-based cardiac rehabilitation" OR "rehabilitation" OR "cardiac-rehabilitation" OR "exercise" OR "exercise therapy")

12. (#4 OR #5 OR #6 OR #7 OR #8 OR #9 OR #10 OR #11 OR #11)

13. (#3 AND #12)

**COCHRANE CENTRAL REGISTER OF CONTROLLED TRIALS**

#1 MeSH descriptor: “Heart transplantation” explode all trees

#2 MeSH descriptor: “Cardiac Rehabilitation” explode all trees

#3 MeSH descriptor: “Telerehabilitation” explode all trees

#4 MeSH descriptor: “Physical education and training” explode all trees

#5 "Heart transplantation" OR "heart transplant" OR "heart transplant recipient" OR “heart graft” OR “cardiac transplant” OR “heart recipient” OR “heart transplant”

#6 "Cardiac Rehabilitation" OR “Rehabilitation” OR "Exercise” OR “Exercise therapy" OR "Sports" OR "Physical exertion" OR “Cardiac-rehabilitation” OR “Exercise-based cardiac rehabilitation” OR "Exercise-based cardiac rehabilitation" OR "exercise" OR "exercise therapy"

#7 (#1 OR #5) AND (#2 OR #3 OR #4 OR #6)

**ISI - WEB OF SCIENCE**

TS=("Heart transplantation" OR "heart transplant" OR "heart transplant recipient" OR “heart graft” OR “cardiac transplant” OR “heart recipient” OR “heart transplant”)

AND

TS=("Cardiac Rehabilitation" OR “Rehabilitation” OR "Exercise” OR “Exercise therapy" OR "Sports" OR "Physical exertion" OR “Cardiac-rehabilitation” OR “Exercise-based cardiac rehabilitation” OR "Exercise-based cardiac rehabilitation" OR "exercise" OR "exercise therapy")

**SCOPUS**

(("Heart transplantation" OR "heart transplant" OR "heart transplant recipient" OR “heart graft” OR “cardiac transplant” OR “heart recipient” OR “heart transplant”)

AND

("Cardiac Rehabilitation" OR “Rehabilitation” OR "Exercise” OR “Exercise therapy" OR "Sports" OR "Physical exertion" OR “Cardiac-rehabilitation” OR “Exercise-based cardiac rehabilitation” OR "Exercise-based cardiac rehabilitation" OR "exercise" OR "exercise therapy"))

**EMBASE**

("Heart transplantation" OR "heart transplant" OR "heart graft" OR "cardiac transplant" OR "heart recipient")

AND

("Cardiac Rehabilitation" OR “Rehabilitation" OR "Exercise” OR "Cardiac-rehabilitation")
