# Supplementary material for: Effectiveness of Exercise-Based Cardiac Rehabilitation for Heart Transplant Recipients: A Systematic Review and Meta-Analysis
Source: Health Serv Insights. 2023 Mar 22;16:11786329231161482. doi: 10.1177/11786329231161482 (PMC10034295; doi:10.1177/11786329231161482)
Supplement: sj-docx-2-his-10.1177_11786329231161482 – Supplemental material for Effectiveness of Exercise-Based Cardiac Rehabilitation for Heart Transplant Recipients: A Systematic Review and Meta-Analysis [file sj-docx-2-his-10.1177_11786329231161482.docx]

**Online Supplementary Material 2:** Table of studies only included in qualitative synthesis

| **1^st^ Author and Year** | **Study description** |  |
| --- | --- | --- |
| Karapolat 2008 [38] | **Study comparing HB and CB** with 40 patients enrolled, 20 to each group. The mean time after transplant was 14.5 ± 17.21 months for CB and 16.69 ± 17.65 months for HB. Intervention consisted in individualized 3 sessions per week with 8 weeks intervention and follow-up total. In both settings RT was included and the intensity of training was intensity consistent with MICT (60-70% pVO2). The main outcome was pVO2. |  |
| Christensen 2010 [44] | A sub-study of Herman 2011 [31] with emphasis on **mental health**. Patients, setting, intervention and follow-up were the same. The mental health outcome used was HADS. | |
| Nytrøen 2013 [42] | A sub-study of Nytrøen 2012 [14] with emphasis on **cardiac allograft vasculopathy**. Of the 52 patients in the main study, 43 (20 in intervention, 23 in control) were enrolled. Setting, intervention and follow-up were the same. The outcomes were several IVUS measurements. | |
| Yardley 2016 [40] | A sub-study of Nytrøen 2012 [14] with emphasis on **maintenance of effect** of the intervention. Of the 52 patients enrolled in the main study, 41 (21 in intervention, 20 in control) were available for the 5 Y-FU evaluation – 4 years after cessation of the intervention. Setting and intervention were the same. The main outcome was pVO2. | |
| Rolid 2020 [41] | A sub-study of Nytrøen 2019 [22] with emphasis on **maintenance of effect** of the intervention. Of the 81 patients enrolled in the main study, 62 (28 in HIIT, 34 in MICT) were available for the 3 Y-FU evaluation – 2 years after cessation of the intervention. Setting and intervention were the same. The main outcome was pVO2. | |
| Rolid 2020 [43] | A sub-study of Nytrøen 2019 [22] with emphasis on **quality of life**. Patients, setting, intervention and follow-up were the same. The QoL outcomes were SF-36 and VAS. | |
