# Supplementary material for: Effectiveness of Exercise-Based Cardiac Rehabilitation for Heart Transplant Recipients: A Systematic Review and Meta-Analysis
Source: Health Serv Insights. 2023 Mar 22;16:11786329231161482. doi: 10.1177/11786329231161482 (PMC10034295; doi:10.1177/11786329231161482)
Supplement: sj-docx-3-his-10.1177_11786329231161482 – Supplemental material for Effectiveness of Exercise-Based Cardiac Rehabilitation for Heart Transplant Recipients: A Systematic Review and Meta-Analysis [file sj-docx-3-his-10.1177_11786329231161482.docx]

| **EBCR vs UC** | | | | | | | | | | | | | | | | | |
| --- | --- | --- | --- | --- | --- | --- | --- | --- | --- | --- | --- | --- | --- | --- | --- | --- | --- |
|  | | | | **EBCR** | | | | | | |  | **Usual Care** | | | | |  |
|  | | | | **Baseline** | | **Follow-up** | | **∆** | | **Total** |  | **Baseline** | **Follow-up** | **∆** | **Total** | **p value ^a^** | |
|  | **Studies using SF-36** | | | | | | | | | | | | | | | | |
|  | Nytrøen 2012 | Bodily Pain | - | | - | | **-** | | 24 | |  | - | - | - | 24 | NS | |
|  |  | Emot. Performance | - | | - | | **-** | | 24 | |  | - | - | - | 24 | NS | |
|  |  | **Gen. Health** | **-** | | **54** | | **-** | | **24** | |  | **-** | **49** | **-** | **24** | **< 0.05** | |
|  |  | Mental Health | - | | - | | **-** | | 24 | |  | - | - | - | 24 | NS | |
|  |  | Phys. Functioning | - | | - | | **-** | | 24 | |  | - | - | - | 24 | NS | |
|  |  | Phys. Performance | - | | - | | **-** | | 24 | |  | - | - | - | 24 | NS | |
|  |  | Social Functioning | - | | - | | **-** | | 24 | |  | - | - | - | 24 | NS | |
|  |  | Vitality | - | | - | | **-** | | 24 | |  | - | - | - | 24 | NS | |
|  |  | PCS | - | | - | | **-** | | 24 | |  | - | - | - | 24 | NS | |
|  |  | MCS | - | | - | | **-** | | 24 | |  | - | - | - | 24 | NS | |
|  | **Studies using PLC** | | | | | | | | | | | | | | | | |
|  | Tegtbur 2003 ^b^ | Negative mood | - | | - | | **-** | | 20 | |  | - | - | **-** | 12 | NS | |
|  |  | Positive mood | - | | - | | **-** | | 20 | |  | - | - | **-** | 12 | NS | |
|  |  | Psych. function | - | | - | | **-** | | 20 | |  | - | - | **-** | 12 | NS | |
|  |  | **Phys. function** | **-** | | **-** | | **-** | | **20** | |  | **-** | **-** | **-** | **12** | **< 0.05** | |
|  |  | **Phys. well being** | **-** | | **-** | | **-** | | **20** | |  | **-** | **-** | **-** | **12** | **< 0.01** | |
|  |  | Social function | - | | - | | **-** | | 20 | |  | - | - | **-** | 12 | NS | |
|  |  | Social well being | - | | - | | **-** | | 20 | |  | - | - | **-** | 12 | NS | |
|  | **Studies using the** **WHOQoL-BREF** | | | | | | | | | | | | | | | | |
|  | Wu 2008 | Physical | 13.22 ± 1.78 | | 13.84 ± 1.78 | | 0.61 ± 1.70 | | 14 | |  | 14.19 ± 2.33 | 13.64 ± 2.11 | - 0.55 ± 1.27 | 23 | NS | |
|  |  | Psychological | 12.95 ± 1.47 | | 13.33 ± 1.85 | | 0.38 ± 2.00 | | 14 | |  | 14.20 ± 2.13 | 14.00 ± 2.45 | - 0.20 ± 1.01 | 23 | NS | |
|  |  | Social relationship | 14.00 ± 2.85 | | 13.90 ± 2.49 | | 1.00 ± 1.69 | | 14 | |  | 14.49 ± 2.06 | 14.55 ± 1.88 | 0.06 ± 1.30 | 23 | NS | |
|  |  | Environment | 13.57 ± 2.04 | | 14.00 ± 2.30 | | 0.43 ± 1.89 | | 14 | |  | 14.20 ± 2.26 | 14.30 ± 2.1 | 0.11 ± 1.28 | 23 | NS | |
|  | **Studies using VAS scale** | | | | | | | | | | | | | | | | |
|  | Nytrøen 2012 |  | **-** | | **65** | | **-** | | **24** | |  | **-** | **26** | **-** | **24** | **< 0.001** | |
| **HIIT vs MICT** | | | | | | | | | | | | | | | | | |
|  | | | **HIIT** | | | | | | | |  | **MICT** | | | | |  |
|  | | | **Baseline** | | **Follow-up** | | **∆** | | **Total** | |  | **Baseline** | **Follow-up** | **∆** | **Total** | **p value^1^** | |
|  | **Studies using SF-36** | | | | | | | | | | | | | | | | |
|  | Dall 2014 ^c^ | Bodily Pain | - | | 85.2 ± 17 | | **-** | | 16 | |  | - | 83.1 ± 17.2 | **-** | 16 | NS | |
|  |  | Emot. Performance | - | | 89.6 ± 20.1 | | **-** | | 16 | |  | - | 91.7 ± 22.8 | **-** | 16 | NS | |
|  |  | Gen. Health | - | | 65.6 ± 16.4 | | **-** | | 16 | |  | - | 65.5 ± 12.7 | **-** | 16 | NS | |
|  |  | Mental Health | - | | 89.3 ± 7.4 | | **-** | | 16 | |  | - | 89.3 ± 5.8 | **-** | 16 | NS | |
|  |  | Phys. Functioning | - | | 83.1 ± 15.9 | | **-** | | 16 | |  | - | 83.1 ± 15.5 | **-** | 16 | NS | |
|  |  | Phys. Performance | - | | 84.4 ± 25.6 | | **-** | | 16 | |  | - | 83.1 ± 15.5 | **-** | 16 | NS | |
|  |  | Social Functioning | - | | 95.3 ± 11.1 | | **-** | | 16 | |  | - | 96.1 ± 7.5 | **-** | 16 | NS | |
|  |  | Vitality | - | | 77.2 ± 15.5 | | **-** | | 16 | |  | - | 78.4 ± 12.6 | **-** | 16 | NS | |
|  |  | PCS | - | | 89.3 ± 7.4 | | **-** | | 16 | |  | - | 90.0 ± 6.6 | **-** | 16 | NS | |
|  |  | MCS | - | | 76.4 ±11.8 | | **-** | | 16 | |  | - | 77.5 ± 8.9 | **-** | 16 | NS | |
|  | Nytrøen  2019 ^d^ | Bodily Pain | 47.8 ± 9.3 | | 50.5 ± 10.5 | | **2.7 ± 10.0** ^e^ | | 37 | |  | 48.1 ± 9.2 | 49.1 ± 12.2 | **1 ± 11.1** ^e^ | 41 | NS | |
|  |  | Emot. Performance | 46.8 ± 13.1 | | 52.0 ± 9.1 | | **5.2 ± 11.6** ^e^ | | 37 | |  | 50.7 ± 7.7 | 48.7 ± 10.5 | **-2 ± 9.4** ^e^ | 41 | NS | |
|  |  | Gen. Health | 48.2 ± 9.4 | | 50.8 ± 11.0 | | **2.6 ± 10.4** ^e^ | | 37 | |  | 49.8 ± 7.3 | 51.2 ± 9.4 | **1.4 ± 8.6** ^e^ | 41 | NS | |
|  |  | Mental Health | 53.1 ± 11.0 | | 53.7 ± 9.7 | | **0.6 ± 10.4** ^e^ | | 37 | |  | 55.4 ± 7.8 | 54.0 ± 9.7 | **-1.4 ± 8.9** ^e^ | 41 | NS | |
|  |  | Phys. Functioning | 45.0 ± 7.0 | | 50.8 ± 6.0 ^f^ | | **5.8 ± 6.6** ^e^ | | 37 | |  | 46.4 ± 6.4 | 51.6 ± 6.6 ^f^ | **5.2 ± 6.5** ^e^ | 41 | NS | |
|  |  | Phys. Performance | 37.6 ± 10.4 | | 48.1 ± 9.3 ^f^ | | **10.5 ± 9.9** ^e^ | | 37 | |  | 40.8 ± 10.0 | 47.0 ± 10.0 ^f^ | **6.2 ± 10** ^e^ | 41 | NS | |
|  |  | Social Functioning | 46.7 ± 9.9 | | 50.2 ± 9.1 | | **3.5 ± 9.6** ^e^ | | 37 | |  | 48.7 ± 8.7 | 50.7 ± 7.7 | **2 ± 8.2** ^e^ | 41 | NS | |
|  |  | Vitality | 50.6 ± 10.8 | | 52.6 ± 12.7 | | **2 ± 11.9** ^e^ | | 37 | |  | 51.2 ± 9.4 | 53.6 ± 9.0 | **2.4 ± 9.2** ^e^ | 41 | NS | |
|  |  | PCS | 42.8 ± 8 | | 48 ± 9 ^f^ | | **5.3 ± 8.6** ^e^ | | 37 | |  | 43 ± 8 | 49 ± 8 ^f^ | **6 ± 8** ^e^ | 41 | NS | |
|  |  | MCS | 52 ± 13 | | 53 ± 12 | | **1 ± 12.5** ^e^ | | 37 | |  | 55 ± 8 | 52 ± 10 | **-3 ± 9.2** ^e^ | 41 | NS | |
|  | Rolid  2020 ^g^ | PCS | 43 (14) | | 50 (15) | | **-** | | 28 | |  | 44 (9) | 51 (17) | **-** | 34 | NS | |
|  |  | MCS | 59 (13) | | 56 (10) | | **-** | | 28 | |  | 56 (10) | 57 (12) | **-** | 34 | NS | |
|  | **Studies using VAS Scale** | | | | | | | | | | | | | | | | |
|  | Nytrøen 2019 |  | - | | 77 ± 22 | | **-** | | 37 | |  | - | 70 ± 25 | **-** | 41 | NS | |
|  | Rolid 2020^h^ |  | 77 ± 23 | | 76 ± 21 | | **-1 ± 22** ^e^ | | 28 | |  | 72 ± 23 ^i^ | 69 ± 22 | **-3 ± 22.5** ^e^ | 34 | NS | |

**Online Supplementary Material 3**: Table of studies comparing EBCR vs UC and HIIT vs MICT regarding Quality of Life

EBCR: Exercise-Based Cardiovascular Rehabilitation, UC: Usual Care, **∆:** Mean Difference, SF-36: Short Form 36 version 2, Emot: Emotional, Gen: General, Phys: Physical, PCS: Physical Component Summary of SF-36, NS: Non-Significant, MCS: Mental Component Summary of SF-36, PLC: Profile of Quality of Life in the chronically ill, Psych: Psychological, WHOQoL-BREF: Brief version of the World Health Organization Questionnaire on Quality of Life, VAS: Visual Analog Scale, M: Months. FU: Follow-up; ^a^ All the p values reported regard the comparison between groups; ^b^ Values were portrayed schematically in the original article; ^c^ Data not reported in the original article, but in a previous meta-analysis in which the author also participated; ^d^ The majority of the data was from a specific QoL study conducted on the same patients of Nytrøen et al. at 1 Y-FU; ^e^ Values were not given in the original article and were therefore calculated; ^f^ Values were significant (p<0.01) in the same group; ^g^ Involves the same cohort of patients of Nytrøen et al. study, but it is a 3 Y-FU. Data reported using median and interquartile range; ^h^ Involves the same cohort of patients, but it is a 3 Y-FU of Nytrøen et al. study; ^i^ Value at 1 Y-FU and not at baseline.
